# Association of glypican‐6 polymorphisms with lumbar disk herniation risk in the Han Chinese population

**DOI:** 10.1002/mgg3.747

**Published:** 2019-05-20

**Authors:** Baoyang Hu, Wenhua Xing, Feng Li, Zhi Huang, Wenkai Zheng, Demin Ji, Fanglin Niu, Yong Zhu, Xuejun Yang

**Affiliations:** ^1^ Inner Mongolia Medical University Hohhot China; ^2^ Department of thoracolumbar spine surgery The Second Affiliated Hospital of Inner Mongolia Medical University Hohhot China; ^3^ The College of Life Sciences Northwest University Xi'an China

**Keywords:** case–control study, *GPC6* gene, Han Chinese population, lumbar disk herniation, polymorphisms

## Abstract

**Background:**

Lumbar disk herniation (LDH) is a degenerative disorder, which partly results from complex genetic factors. The aim of the study was to investigate the potential associations between glypican‐6 (*GPC6*) variants and LDH risk in Han Chinese population.

**Methods:**

A total of 508 Han Chinese LDH patients and 508 healthy controls were recruited in this study. Six single‐nucleotide polymorphisms (SNPs) in *GPC6* were selected and genotyped using an Agena MassARRAY platform. We used logistic regression method to evaluate the linkage between *GPC6* variants and LDH risk by calculating the odds ratio (OR) and 95% confidence intervals (CIs) in multiple genetic models. HaploReg database was used for SNP functional annotation.

**Results:**

Our result revealed *GPC6* rs4773724 was associated with a decreased risk of LDH in allele model (OR = 0.82, 95% CI: 0.68–0.98, *p* = 0.026), whereas rs1008993 increased the LDH risk (OR = 1.34, 95% CI: 1.05–1.71, *p* = 0.020). Besides, we also found rs4773724 and rs9523981 were associated with susceptibility of LDH among individuals whose age are less than 45. And rs1008993 was associated with increased LDH risk in males. Furthermore, Haplotype analysis showed that the TT (rs4773724, rs1008993) haplotype and GC (rs4773724, rs1008993) haplotype had the opposite effects on the susceptibility of LDH.

**Conclusions:**

For the first time, we suggest that rs4773724 and rs1008993 in *GPC6* were considered as a protective factor and a risk factor for LDH in Han Chinese population, respectively. These results provide new ideas for the treatment and prevention of LDH in Han Chinese population.

## 1 INTRODUCTION

Lumbar disk herniation (LDH) is the most common cause of low back pain, which is complained by more than 80 percent of people during their life, and has led to gradually limit in activity of people under 45 years old [Andersson, 1999]. LDH is known as a multifactorial disease, such as environmental factors can contribute to disk degeneration by altering disk metabolism or mechanical stress. And recent studies have proved that genetic factors had significant impacts accounting for more than 70% individual risk of degenerative disk disease (LDD) (Battie et al., 1995; Sambrook, MacGregor, & Spector, 1999). Additionally, several genetic variations have been determined to be associated with lumbar disk disease in recent years (Annunen et al., 1999; Cong, Zhu, Pang, & Guanjun, 2014; Tilkeridis, Bei, Garantziotis, & Stratakis, 2005). There has a significant interaction between the COL1A1 polymorphism and lumbar disk disease in young military recruits (Tilkeridis et al., 2005). And lumbar disk disease was caused by the different phenotypes that Gln substitutes in the COL2 domain and splices mutation in the COL3 domain of the collagen IX molecule (Annunen et al., 1999). In addition, the interaction between aggrecan gene VNTR polymorphism and obesity play an important role in predicting the occurrence of lumbar disk herniation (Cong et al., 2014). Given the enormous undesirable influence on individual health and social‐economic aspects, exploring the risk contributors of LDH at genetic level is essential to understand the etiology of intervertebral disk (IVD) degeneration and research the prevention and treatment of degenerative disks.

Glypicans (GPCs) are composed of proteoglycans family, which are fixed on the cell surface by a glycosylphosphatidylinositol anchor in the extracellular matrices. In the human genome, six kinds of GPCs have been identified (*GPC1* to *GPC6*), coding products of which are related to the cellular growth control and differentiation. GPCs are interacted on each other (Veugelers et al., 1999). Among them, *GPC6* exerts the function of candidate morphogen cofactors or modulators by regulating unique and developmental expression. Especially, *GPC6* may be associated with LDH by causing osteoporosis (Dequeker, 1997; Im & Kim, 2014) as well as play an important role in cartilage damage by WNT pathway, whose structures are a main part of the endplate (van den Bosch et al., 2015; Daly, Ghosh, Jenkin, Oehme, & Goldschlager, 2016). In addition, GPC6 is also reported to be responsible for LDD by changing the internal environment of nucleus pulposus (NP) cells (Zhou et al., 2017). Thus, we highlighted the importance of *GPC6* in the development of LDH, and speculated that single‐nucleotide polymorphisms (SNPs) of *GPC6* are involved in LDH occurrence.

In the present study, there has been no detailed research of polymorphisms in *GPC6* associated with LDH in the Han Chinese population. Hence, we investigate the roles of *GPC6* polymorphisms related to the risk of LDH in a case–control study. Our study has the further understanding of LDH pathogenesis and provides new strategic ideas for the prevention and treatment of LDH in the future.

## 2 MATERIALS AND METHODS

### 2.1 Ethical statement

The declarations on Helsinki of the World Medical Association have been followed in this study, and informed consents were signed by all participants. This study was conducted with the approval of the Ethics Committee of The Second Affiliated Hospital of inner Mongolia Medical University.

### 2.2 Study subjects

A total of 1,016 Han Chinese individuals were involved in this case–control study. In the case group, 508 patients including 297 men and 211 women were uninterruptedly recruited from The Second Affiliated Hospital of inner Mongolia Medical University in Hohhot from 2015 to 2017. The mean age of the case group was 48.49 ± 13.71 years. During the same period, we recruited 508 volunteers as control group consisting of 297 men and 211 women from the same hospital. The mean age of the control group was 49.16 ± 14.90 years. For a case–control analysis, the case and control group in our research matched in a 1:1 ratio based on gender.

Patients who were involved in the case group meet the following criteria: The Imaging diagnosis method, Clinical signs, and symptoms were applied to diagnosing lumbar disk herniation. Imaging diagnosis method: Pfirrmann grading is a very reliable imaging method for the diagnosis of lumbar disk herniation. It is a grading of the relationship between the disk herniation and the nerve heel. We will include patients with Pfirrmann III and above**.** Clinical signs and symptoms: (a) The leg pain is more severe than the low back pain, and mainly limited to the sciatic nerve or femoral nerve dominating area; (b) The lower limb skin has an abnormal feeling; (c) Lasegue test is positive, the angle is less than the normal 50%; (d) There are two of the following four situations including muscle atrophy, decreased muscle strength, decreased sensation, and decreased tendon reflex. Patients with lumbar spinal stenosis, history of spondylolisthesis, intraspinal tumor, history of spinal fracture, congenital dysplasia of the spine, poliomyelitis, and history of spinal surgery were excluded from this study. All patients had no restrictions on age, gender, or disease grades. Moreover, no patient received any medical treatments within a week before obtaining the blood samples served for the study. All patients were agreed to do standardized neurological examinations, which were performed by two professional spine surgeons. Magnetic resonance imaging (MRI) scans were analyzed by two experienced radiologists to evaluate the specifics of disk herniation.

And 508 healthy individuals were selected as the control samples. To minimize the impact of potential environmental and treatment‐related factors on the results, the exclusion criteria were listed as the follows: back pain, generalized musculoskeletal pain, inflammatory rheumatic disease, diabetic polyneuropathy, cardiovascular disease (NYHA III and IV), cancer, psychiatric disease, alcohol or drug abuse, acute or chronic inflammatory disease, hypertension, diabetes mellitus, and poor DNA quality on blood sample.

### 2.3 Selection of single‐nucleotide polymorphisms

To obtain reliable and significant data, minor allele frequency (MAF) was used as a criterion for SNP selection in this study. All of six SNPs had minor allele frequencies more than 5% in 1,000 genome global populations (http://www.internationalgenome.org/). And we used the HaploReg databases to evaluate the possible functions of six SNPs (https://pubs.broadinstitute.org/mammals/haploreg/haploreg.php).

### 2.4 Genotyping methods

We collected 5 ml peripheral venous blood from each participant, which was kept in tubes with sodium citrate and stored at –80℃ after centrifuging at 2000rpm for 10 min. According to the manufacturer's protocol，genomic DNA was extracted from whole blood samples with by the Gold Mag‐Mini Whole Blood Genomic DNA Purification Kit (Gold Mag Co. Ltd. Xi'an City, China). The purified DNA samples were measured by spectrometry (DU530 UV/VIS spectrophotometer, Beckman Instruments, Fullerton, CA, USA). We designed a Multiplexed SNP Mass EXTEND assay using an Agena MassARRAY Assay Design 2.0 Software (https://agenacx.com/online-tools/). We use an Agena MassARRAY method (Agena, Inc., San Diego, CA) to identify the genotype. PCR reactions in 5 μl are performed, followed by the SAP and iPLEX reaction. The samples are then desalted, dispensed to a SpectroCHIP and analyzed with MALDI‐TOF MS. Agena Typer 4.0 software was performed on analyzing and managing data.

### 2.5 Statistical analysis

The Microsoft Excel 2007, SPSS17.0 statistical software (SPSS, Chicago, IL), and PLINK statistical software (http://zzz.bwh.harvard.edu/plink/ld.shtml) were applied for statistical analysis in this case–control study. The *p* values were two‐sided and *p* < 0.05 was considered as statistically significant differences in all the analyses. An independent sample *t* test and a chi‐square test were used to evaluate differences in the distribution of demographic characteristics between cases and controls. All SNPs were checked for Hardy–Weinberg equilibrium (HWE) using the Fisher's exact test, which was performed by comparing the observed and expected genotype frequencies among control subjects. During the analysis, the distribution of SNP allele and genotype frequencies between cases and controls were compared by a Chi‐squared test or Fisher′s exact test. Associations between glypican‐6 polymorphisms and LDH risk were evaluated by calculating odds ratios (ORs) and 95% confidence intervals (CIs) using logistic regression analysis with adjustment for gender and age. The minor allele of each SNP was assumed to be a risk factor compared to the wild‐type allele. The relationships between *GPC6* polymorphisms and the risk of LDH were evaluated according to four models (dominant, recessive, codominant, and log‐additive models). Finally, the linkage disequilibrium (LD) block construction was performed using the Haploview software package (version 4.2).

## 3 RESULTS

A total of 1016 individuals, including 508 (mean age at: 48.49 ± 13.71) cases and 508 (mean age at: 49.16 ± 14.90) controls, were enrolled in this case–control study. The number of men and women was equal in two groups, which were 297 and 211, respectively. The mean age and the sex were matched in this study. So, there were no significant difference in the basic characteristics between cases and controls. The characteristics of both groups were presented in Table 1.

**Table 1:** Characteristics of cases and controls in this study

Variable(s)	Cases (*n* = 508)	Controls (*n* = 508)	*p* value
Sex *N*(%)			0.064^a^
Male	297	297	
Female	211	211	
Age, year (mean ± *SD*)	48.49 ± 13.71	49.16 ± 14.91	0.090^b^

*p* < 0.05 indicates statistical significance.

a *p* value obtained using Chi‐squared test.

b *p* value obtained from independent sample *t* test.

We found that two SNPs (rs4773724, rs1008993) were associated with the risk of LDH. In Table 2, rs4773724 significantly decreased the risk of LDH (OR = 0.82, 95% CI: 0.68–0.98, *p* = 0.026), and rs1008993 was associated with the increased risk of LDH (OR = 1.34, 95% CI: 1.05–1.71, *p* = 0.020). Basic information was also showed in Table 2 including the possible functions of six SNPs, MAF, HWE, and *p* value. We use the HaploReg to evaluate the expected functions, The MAFs of all SNPs were greater than 5%, and all of these SNPs complied with HWE (*p* > 0.05).

**Table 2:** Basic information of candidate SNPs and SNPs functional annotation in HaploReg v4.1 database in this study

SNP	Alleles	MAF		*p* a value for HWE	OR	95% CI		*p* b	Ref	Alt	SNP functional annotation
	A/B	Case	Control								
rs4773724	G/T	0.361	0.409	0.583	0.82	0.68	0.98	0.026*	G	T	Motifs changed
rs1008993	T/C	0.169	0.132	1.000	1.34	1.05	1.71	0.020*	C	T	DNAse
rs9523981	T/C	0.382	0.349	0.205	1.15	0.96	1.38	0.125	C	T	DNAse, Motifs changed
rs7320969	C/G	0.331	0.341	1.000	0.96	0.79	1.15	0.627	C	G	Motifs changed
rs59624626	G/T	0.278	0.294	0.455	0.92	0.76	1.12	0.404	T	G	DNAse, Motifs changed
rs995810	A/G	0.183	0.186	0.240	0.98	0.78	1.22	0.847	A	G	—

Abbreviations: Alt, Alternation; CI, confidence interval; HWE, Hardy–Weinberg equilibrium; MAF, minor allele frequency; OR, odds ratio; Ref, Reference; SNP, single‐nucleotide polymorphism.

a *p* was calculated by Fisher's exact test.

b *p* was calculated by Chi‐squared test.

* *p* < 0.05 indicates statistical significance.

As shown in Table 3, associations between the SNPs in *GPC6* and the risk of LDH were assessed in four genetic models (codominant, dominant, recessive, and additive). Based on the results, we conclude that rs4773724 and rs1008993 in *GPC6* had statistical significance. Rs4773724 conferred 0.79‐fold, 0.76‐fold, and 0.81‐fold decreased risk of LDH in the codominant model (GG: OR = 0.79, 95% CI: 0.46–0.98, *p* = 0.041), in the dominant model (OR = 0.76, 95% CI: 0.59–0.98, *p* = 0.036), and in the log‐additive model (OR = 0.81, 95% CI: 0.68–0.97, *p* = 0.024). Furthermore, rs1008993 exerted 2.40‐fold, 2.29‐fold and 1.32‐fold increased susceptibility of LDH in the codominant model (TT: OR = 2.40, 95% CI: 1.08–5.36, *p* = 0.032), in the recessive model (OR = 2.29, 95% CI: 1.03–5.08, *p* = 0.042), and in the log‐additive model (OR = 1.32, 95% CI: 1.04–1.68, *p* = 0.023).

**Table 3:** Genotypic model analysis of relationship between SNPs and LDH risk

SNP ID	Model	Genotype	Case	Control	OR (95% CI)	*p* a value
rs4773724	Codominant	T/T	206	174	1.00	0.041*
		G/T	237	252	0.79 (0.60–1.04)	
		G/G	65	82	0.67 (0.46–0.98)	
	Dominant	T/T	206	174	1.00	0.036*
		G/G‐G/T	302	334	0.76 (0.59–0.98)	
	Recessive	G/T‐T/T	443	426	1.00	0.136
		G/G	65	82	0.77 (0.54–1.09)	
	Log‐additive	—	—	—	0.81 (0.68–0.97)	0.024*
rs1008993	Codominant	C/C	356	381	1.00	0.032*
		C/T	132	116	1.21 (0.91–1.63)	
		T/T	20	9	2.40 (1.08–5.36)	
	Dominant	C/C	356	381	1.00	0.061
		C/T‐T/T	152	125	1.30 (0.99–1.72)	
	Recessive	C/T‐C/C	488	497	1.00	0.042*
		T/T	20	9	2.29 (1.03–5.08)	
	Log‐additive	—	—	—	1.32 (1.04–1.68)	0.023*

Abbreviations: CI, confidence interval; HWE, Hardy–Weinberg equilibrium; MAF, minor allele frequency; OR, odds ratio; SNP, single‐nucleotide polymorphism;

a *p* was calculated by unconditional logistic regression adjusted for age and gender.

* *p* < 0.05 indicates statistical significance.

The analysis by age stratification showed that rs4773724 and rs9523981 were associated with the risk of LDH among people younger than 45 years old, as displayed in Table 4. And rs4773724 was associated with a decreased risk of LDH in the codominant model (GT: OR = 0.62, 95% CI: 0.42–0.91, *p* = 0.015), in the dominant model (rs4773724, OR = 0.60, 95% CI: 0.42–0.87, *p* = 0.007) and in the log‐additive model (rs4773724, OR = 0.72, 95% CI: 0.55–0.93, *p* = 0.011). Conversely, rs9523981 was associated with an increased risk of LDH in the codominant model (TT: OR = 1.89, 95% CI: 1.04–3.44, *p* = 0.038) and in the log‐additive model (OR = 1.35, 95% CI: 1.03–1.77, *p* = 0.033). However, there were no connections in these two SNPs and the risk of LDH among people older than 45 years old. Additionally, rs1008993 was associated with LDH risk in men, but not in women (Table 5). Rs1008993 was significantly increased the risk of LDH in the dominant model (OR = 1.51, 95% CI: 1.05–2.18, *p* = 0.027) and in the log‐additive model (OR = 1.47, 95% CI: 1.08–2.01, *p* = 0.016) in Table 5.

**Table 4:** Stratified analysis of *GPC6* polymorphisms by age and risk of LDH

SNP ID	Model	Genotype	<45 years				>45 years			
			Case	Control	OR (95% CI)	*p* avalue	Case	Control	OR (95% CI)	*p* avalue
rs4773724	Codominant	T/T	114	83	1.00	0.015*	92	91	1.00	0.471
		G/T	102	120	0.62 (0.42–0.91)		135	132	1.00 (0.68–1.46)	
		G/G	32	41	0.56 (0.33–0.97)		33	41	0.82 (0.47–1.41)	
	Dominant	T/T	114	83	1.00	0.007*	92	91	1.00	0.806
		G/G‐G/T	134	161	0.60 (0.42–0.87)		168	173	0.96 (0.67–1.37)	
	Recessive	G/T‐T/T	216	203	1.00	0.210	227	223	1.00	0.432
		G/G	32	41	0.73 (0.44–1.20)		33	41	0.82 (0.50–1.35)	
	Log‐additive	—	—	—	0.72 (0.55–0.93)	0.011*	—	—	0.93 (0.72–1.20)	0.559
rs9523981	Codominant	C/C	83	100	1.00	0.038*	106	108	1.00	0.816
		T/C	129	121	1.29 (0.88–1.89)		121	123	0.98 (0.67–1.41)	
		T/T	36	23	1.89 (1.04–3.44)		33	32	1.07 (0.61–1.87)	
	Dominant	C/C	83	100	1.00	0.083	106	108	1.00	0.966
		T/C‐T/T	165	144	1.39 (0.96–2.00)		154	155	0.99 (0.70–1.41)	
	Recessive	T/C‐C/C	212	221	1.00	0.085	227	231	1.00	0.762
		T/T	36	23	1.63 (0.93–2.85)		33	32	1.08 (0.64–1.83)	
	Log‐additive	—	—	—	1.35 (1.03–1.77)	0.033*	—	—	1.02 (0.79–1.31)	0.906

Abbreviations: CI, confidence interval; HWE, Hardy–Weinberg equilibrium; MAF, minor allele frequency; OR, odds ratio; SNP, single‐nucleotide polymorphism.

a *p* was calculated from Wald test adjusted for age and gender.

* *p* < 0.05 indicates statistical significance.

**Table 5:** Stratified analysis of *GPC6* polymorphisms by sex and risk of LDH

SNP ID	Model	Genotype	Male				Female			
			Case	Control	OR (95% CI)	*p* a value	Case	Control	OR (95% CI)	*p* a value
rs1008993	Codominant	C/C	205	229	1.00	0.054	151	152	1.00	0.214
		T/C	79	62	1.42 (0.97–2.09)		53	54	0.99 (0.64–1.5)	
		T/T	13	6	2.43 (0.91–6.52)		7	3	2.39 (0.60–9.42)	
	Dominant	C/C	205	229	1.00	0.027*	151	152	1.00	0.780
		T/C‐T/T	92	68	1.51 (1.05–2.18)		60	57	1.06 (0.69–1.63)	
	Recessive	T/C‐C/C	284	291	1.00	0.109	204	206	1.00	0.212
		T/T	13	6	2.23 (0.84–5.96)		7	3	2.39 (0.61–9.40)	
	Log‐additive	—	—	—	1.47 (1.08–2.01)	0.016*	—	—	1.13 (0.77–1.65)	0.528

Abbreviations: CI, confidence interval; HWE, Hardy–Weinberg equilibrium; MAF, minor allele frequency; OR, odds ratio; SNP, single‐nucleotide polymorphism.

a *p* was calculated from Wald test adjusted for age and gender.

* *p* < 0.05 indicates statistical significance.

**Figure 1:**
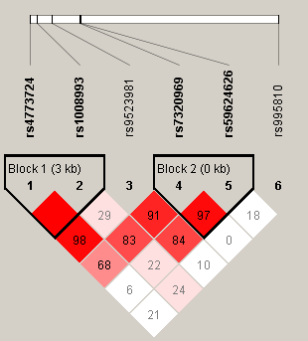
Linkage disequilibrium (LD) analysis of the SNPs on *GPC6* Bright red is a significant LD displayed by standard colors schemes. Parameters r^2^ and D’ were used to analyze LD pattern

Finally, the pairwise linkage disequilibrium (LD) of *GPC6* was analyzed according to confidence intervals method (D’>0.9 and r^2^>0.8). Figure 1 illustrated the existence of two blocks (Block 1: rs4773724 and rs1008993; Block 2: rs7320969 and rs59624626) that had very strong linkage disequilibria. Moreover, logistic regression analysis and Chi‐squared test were used to perform haplotype analysis. There were no statistically significant associations between haplotype comprised rs7320969 and rs59624626 and the risk of LDH. In Table 6, the haplotype “TT” consisted of rs4773724 and rs1008993 was associated with a significantly increased risk of LDH (OR = 1.32, 95% CI = 1.04–1.68, *p* = 0.020). Nevertheless, the haplotype “GC” consisted of rs4773724 and rs1008993 was found to be associated with a decreased risk of LDH (OR = 0.81, 95% CI: 0.68–0.97, *p* = 0.023).

**Table 6:** the haplotype frequencies of *GPC6* polymorphisms and their association with the risk of LDH

Haplotype		Frequency	OR (95% CI)	*p*
rs4773724	rs1008993			
T	T	0.169	1.32 (1.04–1.68)	0.020*
G	C	0.361	0.81 (0.68–0.97)	0.023*
T	C	0.531	0.95 (0.80–1.14)	0.589

Abbreviations: CI, confidence interval; OR, odds ratio.

*p* was calculated using unconditional logistic regression analysis with adjustments for gender and age.

* *p* < 0.05 indicates statistical significance.

## 4 DISCUSSION

LDH is considered to be a disease caused by the multifactorial interactions (Lin et al., 2013; Mu, Ge, Zuo, Chen, & Huang, 2014). To better understand the complex pathogenesis of LDH, we explore the association of GPC6 polymorphisms with LDH risk in the Han Chinese population for the first time. Our findings indicated the special meaning of rs4773724 and rs1008993 in GPC6. Therefore, we speculated that there were certain associations between GPC6 and LDH risk.

At present, it has been extensively studied (Dinccelik‐Aslan, Gumus‐Akay, Elhan, Unal, & Tukun, 2015; Lau et al., 2010; Paine‐Saunders, Viviano, & Saunders, 1999), that GPC6 could be involved in LDH development in many aspects. Firstly, GPC6 expressed in chondrocytes of mouse growth plates, and might induce the degeneration of cartilage endplates and even structural abnormal ossification by WNT and hedgehog signaling pathways. (Campos‐Xavier et al., 2009; Daly et al., 2016; Zhou et al., 2017). Secondly, GPC6 may affect the main components of the nucleus pulposus (NP) by BMP‐7, such as type I and type II collagen (Kim et al., 2010). The abnormal regulation of GPC6 on BMP pathways results in ossification of the annulus and formation of aberrant bone (Kemp et al., 2017; Kim et al., 2010). Finally, Dequeker et al. have reported that osteoporosis had relationship with disk degeneration (Dequeker, 1997; Im & Kim, 2014). They found *GPC6* might regulate osteoblastic bone formation and bone mineral density (BMD) in humans by WNT signaling pathway (Dequeker, 1997). Interestingly, *GPC6* seems to increase the risk of spinal osteoarthritis by causing osteoporosis, and osteoarthritis in the spine increases the levels of inflammatory cytokines such as IL‐1, and TNF‐a, which lead to homeostatic imbalance in disk matrix (Boos et al., 2002). We deemed that GPC6 is a susceptible gene for LDH.

In our study, the minor G allele of rs4773724 could be regarded as a protective factor that decreased the risk of LDH. In contrast, the minor T allele of rs1008993 was associated with an increased risk of LDH. In stratified analysis, our study confirmed the conclusion that rs4773724 and rs9523981 in *GPC6* affect the risk of LDH in the younger population. And rs1008993 had different influence on LDH based on gender. According to the results of haplotype analysis, there were statistically significant association between *GPC6* and LDH risk. As is shown in Table 2, the function of six SNPs was predicted by using HaploReg. The results showed rs4773724 and rs1008993 were involved in the regulations related to motifs changed and DNAse, separately. Although, rs4773724 and rs1008993 were located within intron region of *GPC6*. A number of evidences showed that the extent of splicing mutations was vital. Owing to the disruption of the splice site, mRNA may be influenced by sequence variation occurring in intron, subsequently result in the defects of initiation, elongation, and termination of transcription, which may lead to some diseases (Baralle & Baralle, 2005). Furthermore, we speculate that the variation of *GPC6* intron may be related to the influence of certain genes such as immune regulatory genes, tissue specific genes, and environmental factors (Ban et al., 2012), which may be the key to the variation of *GPC6* SNP and the risk of LDH. Further structural functional studies are required to substantiate this mode. To sum up, rs4773724 and rs1008993 in *GPC6* were associated with the risk of LDH, so *GPC6* variants may be considered as markers in LDH susceptibility assessment for Han Chinese population.

However, our study had several limitations. The specific mechanisms of *GPC6* polymorphisms are still unclear, and our study did not take occupational factors into account. Therefore, further studies including more samples and more detailed information are required to elucidate the association between *GPC6* polymorphisms and the structure of the lumbar disk with its surrounding tissue. We hope these discoveries facilitate understanding of the etiology of LDH and provide a new direction for the prevention and treatment of lumbar disk herniation.

## 5 CONCLUSION

In conclusion, this study reported the potential association of *GPC6* polymorphisms with lumbar disk herniation risk in the Han Chinese population for the first time. Our results revealed that rs4773724 and rs1008993 in *GPC6* were significant associated with the risk of LDH.

## CONFLICT OF INTEREST

No conflict of interest exits in the submission of this manuscript, and manuscript is approved by all authors for publication.
